# Longitudinal Changes in IgG N-glycome Are Associated with Disease Severity and Mortality in COVID-19 Patients

**DOI:** 10.3390/v18090960

**Published:** 2026-09-01

**Authors:** Martina Vinicki, Frano Vučković, Kristian Bodulić, Alemka Markotić, Zoran Barušić, Renata Laškaj, Ivan Christian Kurolt, Sanja Zember, Rok Čivljak, Ivan Puljiz, Gordan Lauc, Tea Pribić

**Affiliations:** 1 Genos Glycoscience Research Laboratory, 10 000 Zagreb, Croatia; 2 University Hospital for Infectious Diseases, 10 000 Zagreb, Croatia; 3 Faculty of Medicine, Catholic University of Croatia, 10 000 Zagreb, Croatia; 4 Faculty of Medicine, University of Rijeka, 51 000 Rijeka, Croatia; 5 School of Medicine, University of Zagreb, 10 000 Zagreb, Croatia; 6 Department of Biochemistry and Molecular Biology, Faculty of Pharmacy and Biochemistry, University of Zagreb, 10 000 Zagreb, Croatia

**Keywords:** IgG glycosylation, COVID-19, mortality, disease severity, biomarkers

## Abstract

Severe COVID-19 is characterized by immune dysregulation, yet reliable biomarkers associated with adverse outcomes remain limited. Immunoglobulin G (IgG) N-glycosylation modulates antibody effector function and reflects the immune system’s inflammatory state. In this study, we investigated whether specific IgG glycosylation profiles are associated with COVID-19 severity, mortality, and clinical course in patients without known prior SARS-CoV-2 infection. Of the 806 admitted COVID-19 patients included in the study, 377 hospitalized patients were longitudinally monitored, and 2001 serum samples were analyzed. Significant alterations in IgG N-glycome composition were observed in patients with severe disease and those who died. The most prominent changes included increased agalactosylation and decreased mono- and digalactosylation, features associated with a more pro-inflammatory IgG glycosylation profile. Nosocomial infections were associated with a more pro-inflammatory IgG glycosylation profile. No statistically significant differences in IgG N-glycome composition were observed between vaccinated and unvaccinated patients, although the small number of vaccinated patients limited this analysis. The observed glycomic profiles likely reflect immune responses characteristic of the early phase of the COVID-19 pandemic. Whether similar glycosylation changes occur in other infectious or inflammatory diseases remains to be established. IgG glycosylation analysis holds promise for personalized risk stratification and early therapeutic intervention in COVID-19 and other severe infectious diseases.

## 1. Introduction

The coronavirus disease 2019 (COVID-19) pandemic, caused by severe acute respiratory syndrome coronavirus-2 (SARS-CoV-2), remains a major global health challenge. Characterizing the epidemiological features and elucidating the pathogenic mechanisms of COVID-19 are critical for disease management and improved patient outcomes [1]. Clinical manifestations of COVID-19 vary widely, ranging from mild symptoms to severe, life-threatening disease. Severe cases and poor outcomes are more prevalent among older adults, individuals with comorbidities, unvaccinated patients, and those infected with particular viral variants [2]. While the respiratory tract is the primary site of SARS-CoV-2 infection, COVID-19 is increasingly recognized as a systemic disease. One of the main drivers of severe disease is excessive immune activation and the overproduction of pro-inflammatory cytokines, a phenomenon closely linked to negative clinical trajectories. Thus, beyond clinical assessment, laboratory identification of immune dysregulation is vital for timely and targeted immunomodulatory therapy [3].

Protein glycosylation, a common and complex post-translational modification, is fundamental to numerous cellular processes, including protein stability, trafficking, clearance, and immunogenicity [4]. In the immune system, glycosylation is particularly important for the function of immunoglobulin G (IgG), a glycoprotein central to host defense against pathogens. The nature of the immune response mediated by IgG antibodies depends on the interaction between Fc receptors and different receptors on the surface of immune effector cells [5]. These interactions can activate several immune processes, such as complement-dependent cytotoxicity (CDC), antibody-dependent cell-mediated cytotoxicity (ADCC), or phagocytosis. Depending on the type of immune response, IgG can exhibit pro-inflammatory or anti-inflammatory effects [6]. IgG glycosylation is a key determinant influencing the outcome of these immune processes. Consequently, IgG glycans show great potential as biomarkers for certain pathological and physiological conditions, such as infection, cancer, and aging [7].

Several studies have investigated the influence of the IgG N-glycome on COVID-19 progression. Vicente et al. reported a correlation between deficient IgG galactosylation and poor COVID-19 outcomes [8]. Larsen et al. identified a positive association between afucosylated IgG, excessive pro-inflammatory cytokine production, and severe disease manifestations [9]. A study by Chakraborty et al. also pointed toward the negative effects of pro-inflammatory IgG on the course of COVID-19, including the presence of afucosylated IgG1 [10]. Notably, in patients with mild disease or those vaccinated with mRNA vaccines, IgG antibodies tend to be more fucosylated and sialylated, paralleling a reduction in pro-inflammatory activity [10]. In addition, antibodies induced by the vaccine were significantly enriched in fucose and sialic acids, both of which were ultimately associated with a reduction in the pro-inflammatory effects of IgG [11]. Pongracz et al. conducted a prospective study analysing IgG1 glycome in hospitalized COVID-19 patients. This study indicated that low galactosylation, low sialylation, and high bisection are linked to a more severe course of COVID-19 [12]. Similar results were reported by Petrović et al., who demonstrated a positive correlation between COVID-19 severity, low IgG galactosylation, and high IgG bisection in different European patient cohorts [13].

Despite these findings, the interplay between IgG N-glycosylation, disease severity, and commonly measured biochemical parameters in larger cohorts remains unexplored. Moreover, the influence of vaccination, SARS-CoV-2 variants, and nosocomial infections on IgG N-glycosylation during COVID-19 has yet to be fully elucidated. These observations underscore the potential of IgG N-glycosylation profiles not only as markers of immune dysregulation but also as biomarkers for adverse outcomes and mortality in COVID-19. In this context, our study aims to provide deeper insight into the longitudinal dynamics of IgG N-glycosylation and its associations with disease severity and outcome, relevant biochemical parameters, vaccination status, and viral variants in hospitalized patients with COVID-19.

## 2. Materials and Methods

### 2.1. Test Subjects and Sample Collection

This longitudinal retrospective study analysed 2001 serum samples from 806 COVID-19 patients admitted to the University Hospital for Infectious Diseases, Zagreb, the leading institution for the treatment of COVID-19 in Croatia. Samples were randomly selected from the admitted patients during two pandemic waves: November–December 2020 and March–July 2021. Of the 806 admitted COVID–19 patients, 377 (46.8%) were longitudinally monitored and contributed 2–18 serial samples. Patients who were not longitudinally monitored contributed only their first available sample to the baseline analyses. Baseline was defined as the first available blood sample obtained from each patient, regardless of the day of disease onset. For longitudinal analyses, Day 0 was defined as the day of disease onset. The time of each sample relative to disease onset was calculated as the interval between the sampling date and the hospital admission date, plus the day of disease recorded at the first clinical assessment. Subsequent samples were obtained during hospitalization as part of routine clinical care whenever blood sampling was clinically indicated. Therefore, both the number and timing of samples varied between patients. All recruited patients had a confirmed SARS-CoV-2 infection, as determined by RT-PCR of nasopharyngeal swab samples. Demographic and epidemiological data were retrieved for all admitted patients. Biochemical and hematological parameters available from routine clinical laboratory evaluation were analyzed and included C-reactive protein (CRP), procalcitonin, albumin, sodium, potassium, chloride, glucose, liver enzymes, bilirubin, serum creatinine, urea, hemoglobin, and complete blood count. All available biochemical and hematological parameters were considered in the analyses using the same statistical framework and were not pre-specified as individual primary biochemical outcomes. For clarity, only biochemical and hematological parameters showing statistically significant associations with IgG N-glycome measures after adjustment for multiple testing are presented in the main manuscript. Clinical parameters were collected from hospitalized patients’ medical records following standard ethical guidelines. Disease severity was determined according to the most severe disease stage reached during hospitalization, based on the national treatment guidelines, as shown in Supplementary Table S1. The national COVID–19 treatment guidelines include severity classification criteria for both adult and pediatric patients and classify immunocompromised patients one severity category higher. Viral lineages were not confirmed through sequencing. Instead, variant assignment relied on the predominant circulating SARS-CoV-2 variant during each epidemic period, as distinct pandemic waves corresponded to specific variants present in the population (November–December 2020: B.1 variant; March–July 2021: B.1.1.7 variant) [14]. This research was approved by the Ethics Committee of the University Hospital for Infectious Diseases (protocol code 01-167-1-2020, date of approval: 29 April 2021).

Blood samples were collected in EDTA-coated vacuum tubes and processed within 1 h: samples were centrifuged at 1620× *g* for 10 min, serum aliquots transferred to 2 mL tubes, centrifuged at 2700× *g* for 10 min, and stored at −20 °C until further analysis.

### 2.2. IgG Isolation

Samples were distributed across 22 96-well plates. IgG was isolated using the CIM^®^ r-Protein G LLD 0.2 mL Monolithic 96-well Plate (BIA Separations, Ajdovscina, Slovenia), following a modified high-throughput protocol adapted from previous methods [15]. IgG was eluted using 0.1 M formic acid (pH 2.5, prepared in-house) and neutralized with 1 M ammonium bicarbonate (Acros Organic, Fair Lawn, NJ, USA). The monolithic plate was stored post-use in 20% (*v*/*v*) EtOH in 20 mM TRIS + 0.1 M NaCl, pH 7.4, at 4 °C. Aliquots of the IgG eluate (average 15 μg) were transferred to PCR plates (Thermo Scientific, Hemel Hempstead, UK) and dried in a vacuum centrifuge. IgG N-glycan analysis (by HILIC-UPLC) followed, including deglycosylation, labeling, and clean-up steps with the GlycoWorks RapiFluor-MS N-Glycan Kit (Waters, Milford, MA, USA), adapted for high throughput.

### 2.3. Glycan Release and Labeling

As published previously, the glycan release and labeling procedure involved several steps [16]. In summary, dried IgG eluate (average mass: 15 µg) was reconstituted in ultrapure water, and a 5% Glyco-Works RapiGest SF solution (Waters, Milford, MA, USA) was added to each sample to denature IgG. N-glycans were enzymatically detached from IgG using Glyco-Works Rapid PNGase F (Waters, Milford, MA, USA) and subsequently labeled with Glyco-Works RapiFlour-MS Solution (Waters, Milford, MA, USA). Following labeling, acetonitrile (ACN, Carlo Erba, Barcelona, Spain) was incorporated into the samples. These were promptly transferred to a Glyco-Works HILIC µElution Plate (Waters, Milford, MA, USA) for clean-up via hydrophilic interaction liquid chromatography solid-phase extraction (HILIC-SPE). The elution of glycans was accomplished using Glyco-Works SPE Elution Buffer (Waters, Milford, MA, USA), comprising 200 mmol/L ammonium acetate/ACN (95:5, *v*/*v*) at pH 7 (Waters, Milford, MA, USA). The eluates were then diluted with 310 µL of Glyco-Works Sample Diluent (dimethylformamide/ACN) (Waters, Milford, MA, USA). Subsequently, 40 µL of each sample was transferred into vials and analyzed using high-performance liquid chromatography based on hydrophilic interactions (HILIC-UHPLC), while the remaining samples were stored at −20 °C.

### 2.4. HILIC–UHPLC Analysis

Purified and labeled IgG N-glycans were analyzed using a Waters Acquity UHPLC system (Waters, Milford, MA, USA). The separation temperature was 60 °C, with samples being kept at 10 °C prior to injection. N-glycans were separated on a Waters Glycan BEH Amide column (2.1 × 100 mm, 1.7 μm). The mobile phase consisted of solvent A (50 mM ammonium formate, pH 4.4) and solvent B (acetonitrile). The gradient ran from 75% to 61.5% acetonitrile over 42 min at 0.4 mL/min. Chromatograms were divided into 22 peaks corresponding to previously described glycan structures [17].

### 2.5. Statistical Analysis

To reduce experimental variation, normalization and batch correction were performed on the UPLC glycan data. To ensure comparability across samples, normalization by total area was performed. Before batch correction, normalized glycan measurements were log-transformed due to the right-skewed distributions and the multiplicative nature of batch effects. Batch correction was performed on log-transformed measurements using the ComBat method (R version 4.4.1, package sva, version 3.54.0), where the technical source of variation (which sample was analyzed on which plate) was modeled as the batch covariate. To correct measurements for experimental noise, estimated batch effects were subtracted from log-transformed measurements. Longitudinal analysis of patient samples throughout the observation period was performed using a linear mixed-effects model, where glycan measurement was the dependent variable, analysed patient groups and their interaction were modeled as fixed effects, and time was modeled both as a fixed effect and a random slope, with individual patient ID being included as a random intercept to account for repeated measurements within individuals. The subject-specific random intercept accounts for between-subject variability, including time-invariant participant characteristics. Before the analyses, glycan variables were transformed to a standard normal distribution using inverse rank-based normal transformation (R package GenAbel, version 1.8.0, function rntransform). Using rank-transformed variables makes estimated effects of different glycans comparable, as they have the same standardized variance. The false discovery rate (FDR) was controlled by the Benjamini–Hochberg procedure at the specified level of 0.05. Data were analyzed and visualized using the R programming language (version 4.0.2.) [18].

## 3. Results

### 3.1. Patient Characteristics

IgG N-glycome composition was analysed in 2001 sera from 806 COVID-19 patients admitted to the University Hospital for Infectious Diseases. The main patient characteristics are shown in Table 1. The median number of sera per patient was 1 (IQR: 1−3. range: 1−18), with one serum being available for 429 (53.2%) patients. Two sera were available for 168 (20.8%) patients, and three or more sera for 209 (25.9%) patients. Overall, 64.1% of patients were male with a median age of 60.1 years (IQR: 47.5–69.7 years, range: 0.1−94.7 years). Furthermore, 50.1% of patients were admitted between November and December 2020, during the predominance of the B.1 viral variant (early SARS-CoV-2 lineage), while 49.9% were admitted between March and July 2021, corresponding to the B.1.1.7 variant. Additionally, 5.7% of patients received at least one mRNA vaccine dose. The median number of comorbidities was 2 (IQR 1–3; range 1−11), with the most common comorbidities being hypertension (36.7%), obesity (15.0%), diabetes (12.4%), and heart disease (7.2%). When considering disease severity on admission, 39.7% of patients had mild COVID-19, 14.1% had moderate disease severity, 35.4% had severe COVID-19, and 10.8% had critical COVID-19. Furthermore, 255 (31.6%) patients suffered from nosocomial infections during hospitalization. A total of 72 (8.9%) analysed patients died.

### 3.2. Association Between IgG N-glycome, COVID-19 Severity and Outcome

**Figure 1:**
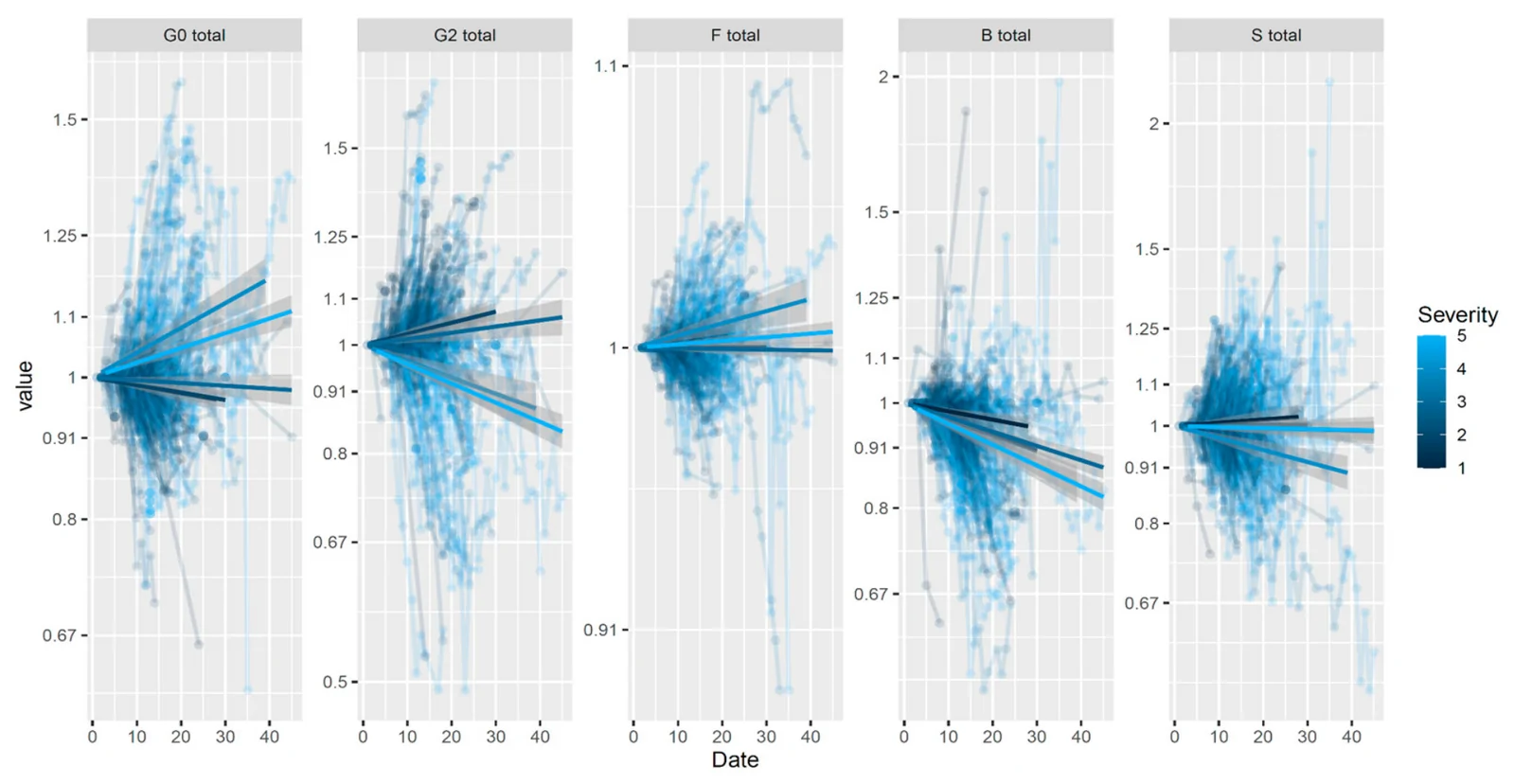
Relative changes in IgG N-glycome in COVID-19 patients grouped by outcome and severity. The bold line represents the model-estimated trajectory for each patient group, with shaded areas indicating the corresponding 95% confidence intervals. 1 = survivors with mild COVID-19, 2 = survivors with moderate COVID-19, 3 = survivors with severe COVID-19, 4 = survivors with critical COVID-19, 5 = deceased COVID-19 patients. G0 = agalactosylated N-glycans, G1 = monogalactosylated, G2 = digalactosylated, F = core-fucosylated, B = bisecting GlcNAc, S = sialylated N-glycans. Date represents the number of days since disease onset, with Day 0 corresponding to the day of disease onset.

To assess the association of COVID-19 severity and clinical outcome with specific glycan traits, we performed a comprehensive analysis of IgG N-glycome composition in both surviving and deceased COVID-19 patients, as well as across groups with different disease severity. Differences in glycan trait trends over time (denoted as effect) were calculated and analyzed. The statistical analysis of changes in IgG N-glycome composition in surviving and deceased COVID-19 patients reveals distinct profiles in specific glycan traits. Compared with survivors, deceased patients showed a significant increase in agalactosylated glycans (G0; effect: 0.0396, p.adj < 0.001) and significant decreases in both monogalactosylated (G1; effect: −0.0357, p.adj = 0.001) and digalactosylated (G2; effect: −0.0573, p.adj < 0.001) glycans, indicating a marked shift toward a pro-inflammatory IgG glycosylation profile. Trends in core fucosylation (F; effect: 0.0195, p.adj = 0.0781) and bisecting GlcNAc (B; effect: −0.0189, p.adj = 0.0781) were not statistically significant after correction for multiple testing, while sialylation (S; effect: 0.0034, p.adj = 0.7782) also showed no significant difference between groups (Table 2). These dynamic profiles, illustrated in Figure 1, show a progressive divergence in glycan traits over time between survivors and non-survivors, with changes in galactosylation being the most pronounced. Taken together, these findings indicate that reduced IgG galactosylation is associated with increased COVID-19 severity and mortality, highlighting its potential as a biomarker of adverse clinical outcomes.

Analysis of patients grouped according to COVID-19 severity revealed similar glycomic profiles. Patients with more severe disease exhibited a significant increase in G0 (effect: 0.0207, p.adj < 0.001) and significant reductions in both G1 (effect: −0.0184, p.adj < 0.001) and G2 (effect: −0.0244, p.adj < 0.001) glycans, again indicating a pro-inflammatory glycan shift with increasing severity. Changes in F (effect: 0.0064, p.adj = 0.2292), B (effect: −0.0030, p.adj = 0.4821), and S (effect: −0.0071, p.adj = 0.2292) were not statistically significant after multiple testing correction (Table 3). As shown in Figure 1, patients with higher disease severity exhibited a more pronounced decline in galactosylated glycans, further supporting the association between IgG N-glycosylation profiles and disease severity in COVID-19.

### 3.3. Association Between IgG N-glycome, SARS-CoV-2 Variant and Patient Vaccination Status

**Figure 2:**
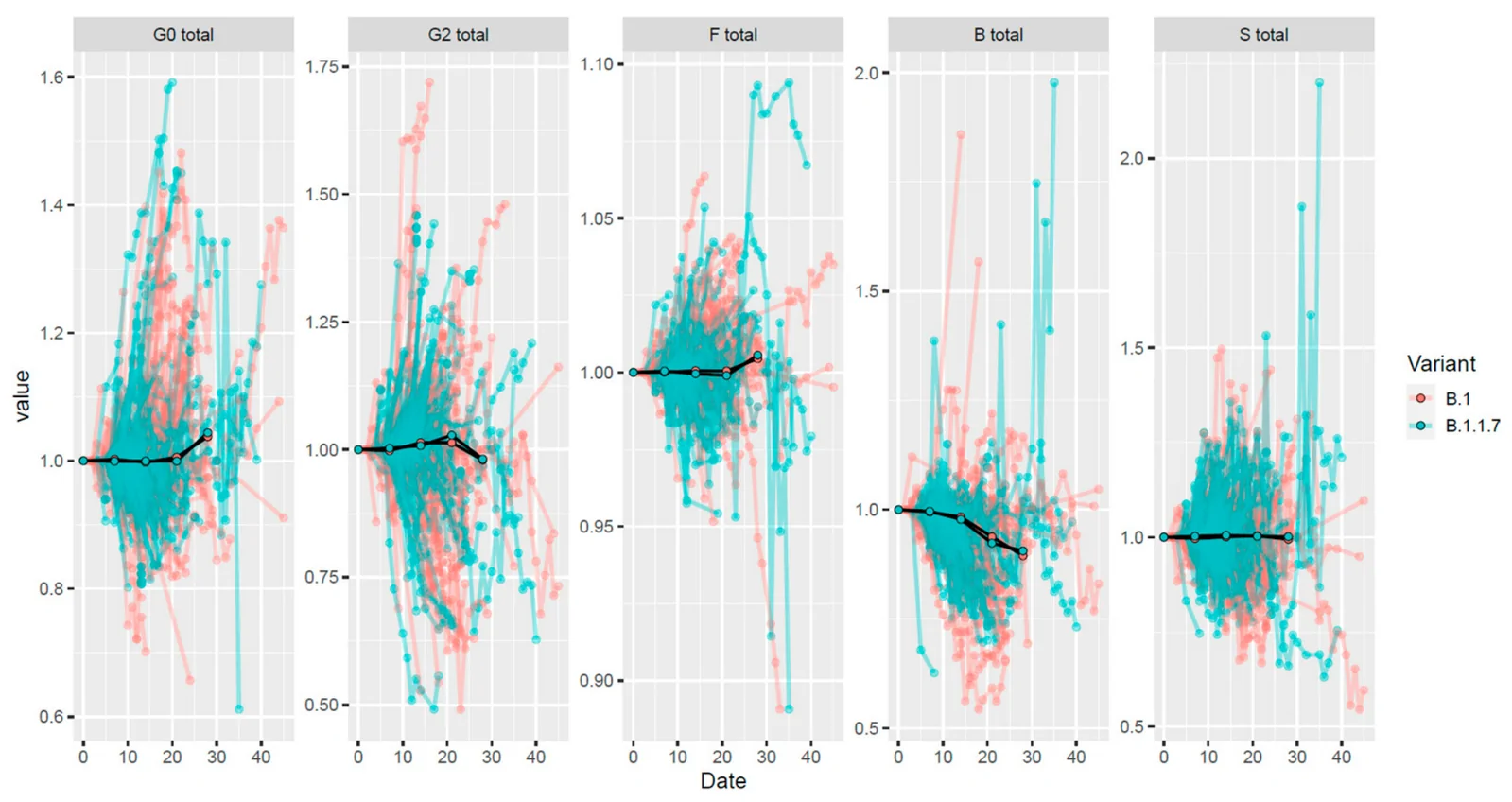
Relative changes in IgG N-glycome traits in COVID-19 patients assigned with the B.1.1.7 and B.1 SARS-CoV-2 variants. The bold black line represents the median value of each glycan trait for each group. G0 = agalactosylated N-glycans, G1 = monogalactosylated N-glycans (one galactose), G2 = digalactosylated N-glycans (two galactoses), F = core-fucosylated N-glycans, B = bisecting GlcNAc, S = sialylated N-glycans. Date represents the number of days since disease onset, with Day 0 corresponding to the day of disease onset.

To assess whether IgG N-glycome profiles differed between major SARS-CoV-2 variants, we compared longitudinal IgG N-glycosylation profiles in patients assigned with the B.1 or B.1.1.7 variants. As shown in Table 4 and Figure 2, no statistically significant differences were observed in glycan traits after correction for multiple testing. Although bisecting GlcNAc (B) showed a nominally significant decrease in B.1.1.7-infected patients (effect: −0.0173, *p* = 0.0312), this effect was not statistically significant after adjustment (p.adj = 0.1872). Similarly, although sialylation (S) tended to increase in the B.1.1.7 group (effect: 0.0175, *p* = 0.0829), this was also non-significant after correction (p.adj = 0.2487). All other glycan traits, including G0, G1, G2, and F, show no significant differences between variant groups. Overall, no statistically significant differences in the IgG N-glycome were observed between patients assigned to the B.1 and B.1.1.7 groups. Because viral variants were inferred based on the epidemiological period of patient admission rather than confirmed by viral genome sequencing, these findings require cautious interpretation (Figure 2).

**Figure 3:**
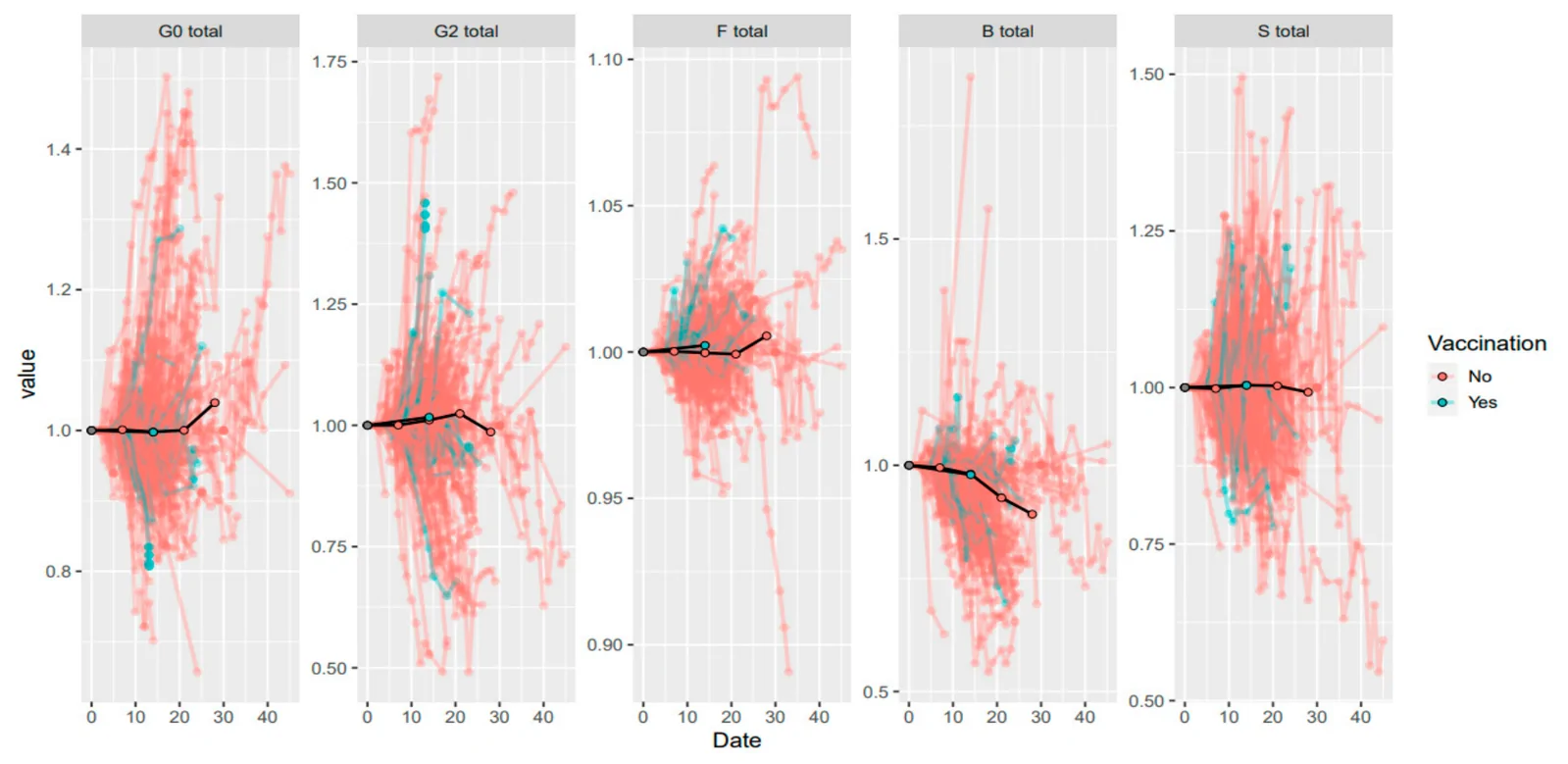
Relative changes in IgG N-glycome traits in vaccinated and unvaccinated COVID-19 patients. The bold black line shows the median value of relative glycan levels for each group. Group 1: unvaccinated; Group 2: vaccinated. G0 = agalactosylated N-glycans, G1 = monogalactosylated N-glycans, G2 = digalactosylated N-glycans, F = core-fucosylated N-glycans, B = bisecting GlcNAc, S = sialylated N-glycans. Date represents the number of days since disease onset, with Day 0 corresponding to the day of disease onset.

Next, we evaluated whether vaccination status affected IgG N-glycome composition in COVID-19 patients. As summarized in Table 5 and Figure 3, vaccinated individuals showed directional changes consistent with a more regulated IgG glycosylation profile, including increased digalactosylation (G2; effect: 0.0114), fucosylation (F; effect: 0.0239), and sialylation (S; effect: 0.0214), and decreased agalactosylation (G0; effect: –0.0071), monogalactosylation (G1; effect: –0.0141), and bisecting GlcNAc (B; effect: –0.0019). However, none of these differences remained statistically significant after correction for multiple testing. The longitudinal trends shown in Figure 3 were consistent with these directional changes; however, no statistically significant differences in the IgG N-glycome composition were observed between vaccinated and unvaccinated patients in this cohort. Given the limited number of vaccinated patients, these findings should be interpreted with caution.

### 3.4. Association Between IgG N-glycome and Nosocomial Infections

We also observed significant changes in the IgG N-glycome associated with nosocomial infections in COVID-19 patients. Of the 806 patients, 255 (31.6%) developed at least one nosocomial infection, accounting for 436 infection episodes. Most affected patients experienced a single episode (*n* = 130), whereas 80, 38, and 8 patients developed two, three, and four episodes, respectively. Sepsis was the most frequent infection category (138 episodes, 31.7%), followed by catheter-associated urinary tract infection (101, 23.2%), ventilator-associated pneumonia (76, 17.4%), and septic shock (40, 9.2%). The remaining episodes included bacterial pneumonia, tracheobronchitis, non-catheter-associated urinary tract infection, enterocolitis, and skin infections. Among episodes with an identified pathogen (*n* = 399), 95.3% were caused by bacteria. Gram-negative organisms predominated, with frequently identified pathogens being multidrug-resistant *Acinetobacter baumannii* (*n* = 189), *Pseudomonas aeruginosa* (*n* = 66), methicillin-resistant *Staphylococcus aureus* (*n* = 32), and ESBL-producing *Enterobacterales*. *Candida parapsilosis* was the only fungal pathogen identified (*n* = 18). The median time of infection onset was 16 days after hospitalization (IQR 10–23), and targeted antimicrobial therapy was administered for a median of 6 days (IQR 3–10). As shown in Table 6, patients who developed nosocomial infections demonstrated a significant increase in agalactosylated IgG glycans (G0; effect: 0.038, *p* < 0.001) and a decreases in both monogalactosylated (G1; effect: –0.033, p.adj < 0.001) and digalactosylated (G2; effect: –0.045, p.adj < 0.001) IgG glycans. All these associations remained statistically significant after correction for multiple testing, indicating a pronounced shift toward a more pro-inflammatory IgG N-glycome in the setting of secondary (hospital-acquired) infections. Other glycan traits, such as core fucosylation (F), bisecting GlcNAc (B), and sialylation (S), showed directional trends but did not reach statistical significance. Importantly, when analyzing stratified data by outcome (survivors vs. deceased), these associations with nosocomial infection were not significant, suggesting that N-glycome alterations may be more closely associated with the presence of secondary infection (mostly bacterial) than with mortality status.

### 3.5. Association Between IgG N-glycome and Selected Biochemical Parameters

We next investigated associations between baseline IgG N-glycome composition and laboratory biochemical parameters (Table 7). Notably, increased baseline G0 (effect: 0.0011, p.adj = 0.043) and decreased G1 (effect: −0.0015, p.adj = 0.009), and G2 (effect: −0.0017, p.adj < 0.001) levels were significantly associated with higher concentrations of CRP, urea, and bilirubin, as well as with lower albumin levels (all p.adj < 0.05). Additionally, bisecting GlcNAc (B) exhibited a significant positive association with albumin and significant negative associations with urea and bilirubin. Furthermore, increased G0, decreased G1 and G2, and reduced B were associated with a higher alkaline phosphatase (ALP). Collectively, these findings indicate that a pro-inflammatory IgG N-glycosylation profile, characterized by elevated G0 and reduced G1 and G2, is associated with laboratory markers of systemic inflammation, impaired liver function, and poor nutritional status.

We also analyzed associations between changes in IgG N-glycome composition and corresponding changes in key biochemical markers (Table 8). In particular, changes in CRP, procalcitonin, and ALP during hospitalization were significantly associated with changes in glycan traits. Specifically, increases in CRP levels were associated with higher G0 (effect: 0.9170, p.adj = 0.009), and lower G1 (effect: –0.9280, p.adj = 0.006), and G2 (effect: –1.3747, p.adj < 0.001) levels. Similar associations were observed for procalcitonin and ALP.

## 4. Discussion

This study presents a comprehensive longitudinal analysis of IgG N-glycome composition in patients without known prior SARS-CoV-2 infection, and its association with disease severity and mortality, as well as viral variants, vaccination status, nosocomial infections, and relevant biochemical parameters. By analyzing 2001 serum samples from 806 hospitalized patients across two pandemic waves, we demonstrated that patients with severe disease or fatal outcomes exhibit a pronounced pro-inflammatory shift in IgG N-glycans, characterized by increased agalactosylation and decreased mono- and digalactosylation.

Our findings are consistent with previous reports linking low IgG galactosylation and, in some studies, afucosylation with worse COVID-19 outcomes [8,11]. A recent comparative study of influenza and COVID-19 showed stable galactosylation in influenza and COVID-19 survivors but a marked decrease in deceased COVID-19 patients, supporting the prognostic value of decreased galactosylation in COVID-19 mortality [19]. These glycosylation changes are mechanistically relevant, as agalactosylated IgG enhances pro-inflammatory immune effector functions, primarily through complement activation and modulation of Fc receptor binding [20,21]. Furthermore, since galactose residues serve as precursors for sialic acid addition, agalactosylation indirectly reduces IgG’s anti-inflammatory potential. Petrović et al. also reported a consistent decrease in galactosylation as disease severity increased [13]. Bisecting GlcNAc showed a nominal decrease with increasing severity, aligning with observations from Larsen et al. in anti-SARS-CoV-2 IgG [9] and Petrović et al. [22]. Another study reported decreased bisecting GlcNAc levels in both influenza and COVID-19 infections [19]. The underlying mechanisms, possibly involving modulation of fucosylation and Fcγ receptor activity, remain to be fully elucidated. Fucosylation trends vary among viruses. While antigen-specific IgG afucosylation is implicated in increased antibody-dependent cellular cytotoxicity (ADCC) and severe disease in other viral infections, total IgG fucosylation remained relatively stable in COVID-19 [19]. Pongracz et al. found no significant difference in fucosylation between ICU and non-ICU hospitalized patients [12]. This aligns with other reports showing that while lower antigen-specific IgG fucosylation correlates with responses to enveloped viruses and infection severity, total IgG fucosylation is stable during COVID-19 [9,10]. In our study, no statistically significant changes in total IgG fucosylation were observed. Hou et al. reported that severe COVID-19 cases had lower total IgG fucosylation than healthy controls, but higher levels than mild cases [23]. Higher bisecting GlcNAc levels on IgG were reported to indirectly affect affinity for FcγRs and enhance ADCC by inhibiting fucosylation [24]. This divergence may reflect differing cytokine milieus, as influenza more robustly activates interferon pathways linked to glycan remodeling, while SARS-CoV-2 is known to dysregulate type I/II interferon cascades. The massive release of pro-inflammatory cytokines, including type I interferons (IFNs), leads to a cytokine storm that contributes to fatal COVID-19 outcomes [25]. Type I IFNs, particularly IFN-α and IFN-β, activate downstream cytokines such as IL-12 and IFN-γ [25]. While type I IFN signalling generally limits virus-induced inflammation, its dysregulation in severe SARS-CoV-2 infection impairs the development of adaptive immunity and is associated with worse clinical outcomes [26]. These immune dysregulation pathways intersect with changes in IgG glycosylation and may underlie variability in glycan profiles and clinical severity among patients. Understanding the interplay between cytokine signaling and antibody glycosylation could provide important insights into COVID-19 pathogenesis and inform therapeutic strategies.

We also explored associations between IgG N-glycome composition and routine biochemical parameters. Strong associations were observed between baseline glycan traits and inflammation markers (CRP and procalcitonin), liver and kidney function (bilirubin, ALP, urea), and nutritional status (albumin). Elevated inflammatory markers were significantly associated with increased agalactosylation and reduced galactosylation and bisecting GlcNAc, reflecting the pro-inflammatory properties of IgG [27]. This effect is partly mediated via complement activation [21]. Conversely, higher albumin levels, indicative of better nutritional and systemic status, are associated with a more anti-inflammatory IgG N-glycosylation profile. Longitudinal changes in IgG N-glycosylation also tracked fluctuations in CRP, procalcitonin, and ALP, underscoring the dynamic interplay between immune glycosylation and clinical biomarkers. These findings support the utility of IgG N-glycans as dynamic biomarkers of immune and physiological status [8].

Beyond severity and mortality, our study found no statistically significant differences in IgG N-glycome between patients assigned to the B.1 and B.1.1.7 groups or between vaccinated and unvaccinated patients. However, SARS-CoV-2 variants were inferred from the epidemiological period of patient admission rather than confirmed by viral genome sequencing. Therefore, these findings should be interpreted with caution and should not be considered evidence that viral lineage has no effect on IgG N-glycosylation. Vaccinated individuals exhibited trends toward increased digalactosylation, fucosylation, and sialylation, although these were not statistically significant, likely reflecting the limited number of vaccinated patients in this study. These findings are consistent with previous studies reporting vaccination-induced IgG glycosylation and highlight the complexity of immune regulation during active infection [10]. Notably, the number of vaccinated patients in our cohort was relatively low, and none had received booster doses, reflecting the time frame of sample collection. As such, the effects of vaccination on IgG N-glycosylation warrant further investigation.

A novel finding is the marked pro-inflammatory glycan shift observed in patients with nosocomial infections. These findings highlight the potential contribution of secondary infections to immune dysregulation and their association with altered IgG N-glycosylation in hospitalized patients with COVID-19. Most nosocomial infections, particularly those occurring in intensive care units, are caused by bacteria. Glycosylation plays a vital role in these infections by supporting bacterial survival and modulating the host immune response. Bacteria use their own glycosylation systems to adhere to host cells, evade immunity, and deliver virulence factors, while also altering host glycosylation profiles to promote infection. Because bacterial glycosylation pathways often differ from those in humans and/or in viral infections, understanding these host–pathogen glycan interactions is essential for developing new anti-infective strategies [28,29,30]. Further research into glycosylation processes during initial viral infections and subsequent bacterial co-infections in hospital settings is of high importance.

Collectively, our results demonstrate that IgG N-glycosylation is associated with immune homeostasis in COVID-19, with a dynamic shift toward a more pro-inflammatory IgG N-glycosylation profile being observed in patients with severe disease, fatal outcomes, and secondary bacterial infections, which frequently complicate severe viral illness. Glycomic profiling holds promise for risk stratification, longitudinal monitoring, and therapeutic guidance in COVID-19 and other systemic inflammatory diseases [31].

While these findings provide important insights into longitudinal changes in IgG N-glycosylation during COVID-19, several limitations warrant consideration. First, serial blood sampling was dictated by routine clinical care rather than a standardized schedule. As a result, patients with prolonged hospital stays contributed more than those discharged early or those who died soon after admission. Second, repeated measurements within individuals were appropriately accounted for, however, age and sex were not included as fixed-effect covariates, and residual confounding by comorbidities cannot be excluded because these variables were not included in the present analyses. Furthermore, analyses of nosocomial infection were not adjusted for important clinical factors, including disease severity, ICU admission, duration of hospitalization, and mechanical ventilation, which may have influenced the observed associations. Third, although the median timing of nosocomial infection onset was available, the timing of infection onset relative to individual blood sample collection was insufficiently detailed. Consequently, we could not determine whether the observed N-glycosylation changes preceded or followed either clinical deterioration or the onset of nosocomial infection, limiting the interpretation of these associations. Finally, the analysis focused on total IgG rather than on SARS-CoV-2-specific antibodies, and neither antigen-specific nor Fc-site-specific glycopeptide analyses were performed. Therefore, direct mechanistic conclusions regarding Fcγ receptor engagement, complement activation, or the functional properties of SARS-CoV-2-specific antibodies cannot be drawn. It is important to note that the population analyzed in this study was recruited during the early pandemic waves and consisted predominantly of patients with no known prior SARS-CoV-2 infection. Therefore, the observed IgG N-glycosylation profiles provide insight into immune responses during the early phase of the COVID-19 pandemic. Whether similar glycosylation changes occur in other infectious or inflammatory diseases remains to be established in future comparative studies. Understanding these early immunoglycomic signatures may be crucial for identifying patients at risk of severe disease and informing timely therapeutic interventions in emerging infectious threats.

In conclusion, this study demonstrates that IgG N-glycosylation profiles, particularly galactosylation, are associated with COVID-19 severity and mortality. Our findings highlight the association between antibody glycosylation and systemic immune dysregulation during COVID-19 and support further investigation of IgG N-glycosylation as a potential biomarker of disease severity and clinical outcome.

## Figures and Tables

**Table 1:** Demographic and clinical characteristics of the analysed COVID-19 patients.

Parameter	N (%)/Median (IQR)
Sex (male)	517 (64.1%)
Age (years) *	60.1 (47.5−69.7)
Age group (years) *	
<18	68 (8.4%)
18−30	19 (2.4%)
31−50	136 (16.9%)
51−65	259 (32.1%)
66−80	223 (27.7%)
>80	58 (7.2%)
Pandemic wave	
November 2020–December 2020 (B.1 variant)	404 (50.1%)
March 2021–July 2021 (B.1.1.7 variant)	402 (49.9%)
Vaccination status	
Unvaccinated	760 (94.3%)
One vaccine dose	3 (0.4%)
Two vaccine doses	43 (5.3%)
Number of comorbidities	2 (1−3)
Disease severity on admission	
Mild	320 (39.7%)
Moderate	114 (14.1%)
Severe	285 (35.4%)
Critical	87 (10.8%)
Nosocomial infection	255 (31.6%)
Day of disease on admission	8 (6−11)
Hospitalization length	10 (7−16)
ICU admission	104 (12.9%)
Mechanical ventilation	68 (8.4%)
Death	72 (8.9%)

* Data unavailable for 43 patients.

**Table 2:** Statistical analysis of IgG N-glycome composition changes in deceased and surviving COVID-19 patients. Effect represents the differences in the longitudinal trend of specific glycan traits between deceased and survivors.

Glycan	Effect	Effect Std. Error	*p*-Value	Adjusted *p*-Value
G0	0.0396	0.0103	<0.001	<0.001
G1	−0.0357	0.0101	<0.001	0.001
G2	−0.0573	0.0118	<0.001	<0.001
F	0.0195	0.0106	0.0651	0.0781
B	−0.0189	0.0010	0.0553	0.0781
S	0.0034	0.0118	0.7782	0.7782

G0 = agalactosylated N-glycans, G1 = monogalactosylated N-glycans, G2 = digalactosylated N-glycans, F = core-fucosylated N-glycans, B = N-glycans with bisecting GlcNAc, S = sialylated N-glycans.

**Table 3:** Statistical analysis of IgG N-glycome composition changes in COVID-19 patients with different disease severity. Effect represents the estimated difference in the rate of change over time for each glycan trait between patients with severe and mild disease forms, as derived from a linear mixed-effects model.

Glycan	Effect	Effect Std. Error	*p*-Value	Adjusted *p*-Value
G0	0.0207	0.0043	<0.001	<0.001
G1	−0.0184	0.0044	<0.001	<0.001
G2	−0.0244	0.0048	<0.001	<0.001
F	0.0064	0.0048	0.1865	0.2292
B	−0.0030	0.0040	0.4821	0.4821
S	−0.0071	0.0054	0.1910	0.2292

G0 = agalactosylated N-glycans, G1 = monogalactosylated N-glycans, G2 = digalactosylated N-glycans, F = core-fucosylated N-glycans, B = N-glycans with bisecting GlcNAc, S = sialylated.

**Table 4:** Statistical analysis of IgG N-glycome composition changes in COVID-19 patients assigned with SARS-CoV-2 B.1.1.7 and B.1 SARS-CoV-2 variants. Effect represents the differences in trends of longitudinal levels of the specific glycan traits between B.1.1.7 and B.1 groups.

Glycan	Effect	Effect Std. Error	*p*-Value	Adjusted *p*-Value
G0	−0.0111	0.0085	0.1964	0.3224
G1	0.0028	0.0085	0.7413	0.7413
G2	0.0118	0.0095	0.2149	0.3224
F	−0.0069	0.0089	0.4447	0.5536
B	−0.0173	0.0080	0.0312	0.1872
S	0.0175	0.0100	0.0829	0.2487

G0 = N-glycans without galactose, G1 = N-glycans with one galactose, G2 = N-glycans with two galactoses, F = N-glycans with core-fucose, B = N-glycans with bisecting GlcNAc, S = sialylated N-glycans.

**Table 5:** Statistical analysis of IgG N-glycome composition changes in vaccinated and unvaccinated COVID-19 patients. Effect represents the differences in longitudinal glycan traits between vaccinated and unvaccinated groups.

Glycan	Effect	Effect Std. Error	*p*-Value	Adjusted *p*-Value
G0	−0.0071	0.0161	0.6614	0.7937
G1	−0.0141	0.0163	0.3876	0.7751
G2	0.0114	0.0178	0.5248	0.7872
F	0.0239	0.0162	0.1401	0.7751
B	−0.0019	0.0151	0.8986	0.8986
S	0.0214	0.0189	0.2604	0.7751

G0 = agalactosylated N-glycans (no galactose), G1 = monogalactosylated N-glycans (one galactose), G2 = digalactosylated N-glycans (two galactoses), F = core-fucosylated N-glycans, B = N-glycans with bisecting GlcNAc, S = sialylated N-glycans.

**Table 6:** Association between IgG N-glycome changes and nosocomial infections in surviving, and deceased patients. Effect represents the difference in the trend of glycan levels between patients with and without nosocomial infections.

	All patients				Survived Patients				Deceased Patients			
Glycan	Effect	St.err	p.val	p.adj	Effect	St.err	p.val	p.adj	Effect	St.err	p.val	p.adj
G0	0.038	0.009	<0.001	<0.001	0.027	0.011	0.016	0.125	0.023	0.025	0.353	0.605
G1	−0.033	0.009	<0.001	0.001	0.021	0.010	0.050	0.200	0.032	0.025	0.201	0.372
G2	−0.045	0.010	<0.001	<0.001	0.028	0.013	0.028	0.167	0.020	0.028	0.493	0.623
F	0.006	0.009	0.532	0.618	0.003	0.012	0.813	0.813	0.030	0.023	0.196	0.372
B	−0.008	0.009	0.376	0.537	0.004	0.011	0.706	0.770	0.016	0.024	0.492	0.623
S	−0.010	0.011	0.338	0.507	0.018	0.001	0.135	0.325	0.001	0.029	0.789	0.813

G0 = agalactosylated N-glycans (no galactose), G1 = monogalactosylated N-glycans (one galactose), G2 = digalactosylated N-glycans (two galactoses), F = core-fucosylated N-glycans, B = N-glycans with bisecting GlcNAc, S = sialylated N-glycans.

**Table 7:** Association between baseline IgG N-glycome levels and selected biochemical parameters.

Glycan	Parameter	Effect	Effect Std. Error	*p*-Value	Adjusted *p*-Value
G0	CRP	0.0011	0.0004	0.006	0.043
G1	CRP	−0.0015	0.0004	<0.001	0.009
G2	CRP	−0.0017	0.0004	<0.001	<0.001
G0	Albumin	−0.0351	0.0086	<0.001	0.001
G1	Albumin	0.0519	0.0068	<0.001	<0.001
G2	Albumin	0.0416	0.0082	<0.001	<0.001
B	Albumin	0.0735	0.0082	<0.001	<0.001
G0	Urea	0.0029	0.0063	<0.001	<0.001
G1	Urea	−0.0339	0.0060	<0.001	<0.001
G2	Urea	−0.0294	0.0068	<0.001	<0.001
B	Urea	−0.0213	0.0065	<0.001	0.009
G0	Bilirubin	0.0097	0.0024	<0.001	<0.001
G1	Bilirubin	−0.0101	0.0026	<0.001	0.001
G2	Bilirubin	−0.0091	0.0023	<0.001	0.001
B	Bilirubin	−0.0089	0.0025	<0.001	0.004
G0	ALP	0.0032	0.0010	0.001	0.011
G1	ALP	−0.0035	0.0011	0.002	0.015
G2	ALP	−0.0035	0.0009	<0.001	0.027
B	ALP	−0.0028	0.0010	0.007	0.050

G0 = agalactosylated N-glycans (no galactose), G1 = monogalactosylated N-glycans (one galactose), G2 = digalactosylated N-glycans (two galactoses), F = core-fucosylated N-glycans, B = N-glycans with bisecting GlcNAc, S = sialylated N-glycans.

**Table 8:** Associations between longitudinal changes in IgG N-glycome and selected biochemical parameters.

Glycan	Parameter	Effect	Effect std. Error	*p*-Value	Adjusted *p*-Value
G0	CRP	0.9170	0.2761	<0.001	0.009
G1	CRP	−0.9280	0.2653	<0.001	0.006
G2	CRP	−1.3747	0.2693	<0.001	<0.001
G0	Procalcitonin	0.9049	0.3690	0.013	0.078
G1	Procalcitonin	−1.1186	0.3244	<0.001	0.006
G2	Procalcitonin	−1.2416	0.3740	<0.001	0.009
G0	ALP	0.9819	0.3700	0.007	0.050
G2	ALP	−1.0354	0.3723	0.005	0.004

G0 = agalactosylated N-glycans (no galactose), G1 = monogalactosylated N-glycans (one galactose), G2 = digalactosylated N-glycans (two galactoses), F = core-fucosylated N-glycans, B = N-glycans with bisecting GlcNAc, S = sialylated N-glycans.

## Data Availability

The datasets generated and analyzed during the current study are available from the corresponding author upon reasonable request.

## Supplementary Materials

The following supporting information can be downloaded at: https://www.mdpi.com/article/10.3390/v18090960/s1.

## Abbreviations

COVID-19	Coronavirus Disease 2019
SARS-CoV-2	Severe Acute Respiratory Syndrome Coronavirus 2
IgG	Immunoglobulin G
N-glycan	N-linked glycan
G0	Agalactosylated IgG glycan
G1	Monogalactosylated IgG glycan
G2	Digalactosylated IgG glycan
F	Core-fucosylated IgG glycan
B	IgG glycan with bisecting N-acetylglucosamine (GlcNAc)
S	Sialylated IgG glycan
Fc	Fragment crystallizable region of IgG
ADCC	Antibody-dependent cellular cytotoxicity
CDC	Complement-dependent cytotoxicity
HILIC	Hydrophilic Interaction Liquid Chromatography
UHPLC	Ultra-High-Performance Liquid Chromatography
EDTA	Ethylenediaminetetraacetic acid
PNGase F	Peptide-N-Glycosidase F
CRP	C-reactive protein
ALP	Alkaline phosphatase
ICU	Intensive Care Unit
FDR	False Discovery Rate
