# Supplementary material for: Longitudinal Changes in IgG N-glycome Are Associated with Disease Severity and Mortality in COVID-19 Patients
Source: Viruses. 2026 Sep 1;18(9):960. doi: 10.3390/v18090960 (PMC13612034; doi:10.3390/v18090960)
Supplement: Supplementary file 1 [file viruses-18-00960-s001.zip › viruses-4468133-supplementary.pdf]

Supplementary Table S1. Criteria for COVID-19 severity classificaiton according to national treatment guidelines employed in this study.

Disease severity*	Criteria
Mild	A patient with symptoms of an uncomplicated respiratory tract infection, including fever, general malaise, headache, myalgia, rhinorrhea, sore throat, and cough.<br><br>A patient without signs of dehydration, sepsis, or respiratory distress (dyspnea).<br><br>A child with an acute upper respiratory tract infection.
Moderate	An adult patient with more severe symptoms of illness and pneumonia, but without criteria for severe pneumonia, and without the need for supplemental oxygen therapy (SpO_2 >93% on room air).<br><br>A child with pneumonia, not meeting criteria for severe disease.
Severe	An adult patient with bilateral pneumonia accompanied by at least one of the following signs: respiratory rate >30 breaths/min, respiratory failure, or the need for supplemental oxygen therapy (SpO_2 ≤ 93% on room air).<br><br>A child with severe pneumonia, presenting with one of the following signs: central cyanosis or SpO_2 ≤ 90%, marked dyspnea, impaired general condition, altered consciousness, or seizures
Critical	An adult patient or child meeting criteria for ARDS**, sepsis, or septic shock, with or without acute dysfunction of other organs (shock, renal failure, coagulopathy).

SpO_2 = Peripheral capillary oxygen saturation, ARDS = Acute respiratory distress syndrome

* Immunocompromised patients should be classified one category higher (more severe disease)

** Definition of ARDS based on PaO_2/FiO_2 ratio: Mild ARDS: PaO_2/FiO_2 200–300 mmHg, Moderate ARDS: PaO_2/FiO_2 100–199 mmHg, Severe ARDS: PaO_2/FiO_2 ≤100 mmHg
